# Catalepsy Produced by a Hair

**Published:** 1878-10

**Authors:** 


Catalepsy Produced by a Hair.—A child in Paris, one year old, in whom no organic disease or other pathological condition could be discovered, was observed for several weeks to have tonic spasms. One day its mother discovered and removed a hair, the end of which was projecting between the two front incisor teeth. This hair, 90 ctm. in length, extended backward, probably into the larynx, its irritation there probably giving rise to the attacks. The disease never returned after its removal.—*Allg. Med. Cent.-Ztg.* No. 68, 1878.

